# Sex Differences in Re-experiencing Symptoms Between Husbands and Wives Who Lost Their Only Child in China: A Resting-State Functional Connectivity Study of Hippocampal Subfields

**DOI:** 10.3389/fnhum.2021.655044

**Published:** 2021-04-28

**Authors:** Yifeng Luo, Yu Liu, Zhao Qing, Li Zhang, Yifei Weng, Xiaojie Zhang, Hairong Shan, Lingjiang Li, Rongfeng Qi, Zhihong Cao, Guangming Lu

**Affiliations:** ^1^Department of Radiology, The Affiliated Yixing Hospital of Jiangsu University, Wuxi, China; ^2^Department of Medical Imaging, Jinling Hospital, Medical School of Nanjing University, Nanjing, China; ^3^Department of Radiology, The Yixing Second Hospital, Wuxi, China; ^4^Department of Radiology, The Affiliated Drum Tower Hospital of Nanjing University Medical School, Nanjing, China; ^5^Key Laboratory of Psychiatry and Mental Health of Hunan Province, Mental Health Institute, The Second Xiangya Hospital, National Technology Institute of Psychiatry, Central South University, Changsha, China

**Keywords:** lost only child, sex differences, couple, functional connectivity, hippocampal subfields, re-experiencing symptom

## Abstract

**Background**: Losing one’s only child may lead to post-traumatic stress disorder (PTSD), of which re-experiencing is the core symptom. However, neuroimaging studies of sex differences in re-experiencing in the context of the trauma of losing one’s only child and PTSD are scarce; comparisons of the functional networks from the hippocampal subfields to the thalamus might clarify the neural basis.

**Methods**: Thirty couples without any psychiatric disorder who lost their only child (non-PTSD group), 55 patients with PTSD, and 50 normal controls underwent resting-state functional magnetic resonance imaging. The functional connectivity (FC) from the hippocampal subregions to the thalamus and the correlations of FC with re-experiencing symptoms were analyzed within and between the sexes.

**Results**: Compared with husbands without PTSD, wives without PTSD had higher re-experiencing symptoms and weaker FC between the right hippocampal cornu ammonis 3 (RCA3) and the right thalamus (RT; RCA3-RT). Moreover, only the correlation between the RCA3-RT FC and re-experiencing in wives without PTSD was significant. Among the three groups, only the RCA3-RT FC in female subjects was markedly different. Additionally, the RCA3-RT FC in wives without PTSD was remarkably lower relative to female patients with PTSD.

**Conclusion**: Wives without PTSD who lost their only child had worse re-experiencing symptoms relative to their husbands, which was associated with the FC alteration between the hippocampal subregions and the thalamus. Importantly, the low level of the RCA3-RT FC may play a potentially protective role against the development of PTSD in wives who have lost their only child.

## Introduction

In China, the one-child policy, legislated in the 1970s, led to millions of single-child families (Hesketh et al., 2005). While this was an effort to curb the growing population, an unintended consequence was that the death of the only child placed parents at a high risk of post-traumatic stress disorder (PTSD) (Li and Wu, 2013; Yin et al., 2018); this included three symptom clusters: re-experiencing, avoidance, and hyperarousal (Association, 2013).

Re-experiencing symptoms, characterized by intrusions of unwanted memories of the traumatic event (Postel et al., 2019), are largely unique to PTSD and are central its diagnosis (Gelernter et al., 2019). Previous studies have reported that deficient context processing could lead to the emergence of recurrent intrusive memories (Liberzon and Abelson, 2016). Thus, the hippocampus, which is known to be involved in contextual memory (Maren et al., 2013), is associated with intrusions. Moreover, the thalamus plays a critical role in perceptual processing and fear conditioning in PTSD (Sherman and Guillery, 2006; Haber and Calzavara, 2009; Zhu et al., 2017). Additionally, there is existing evidence demonstrating that dysfunction within the interconnected context processing circuitry, which involves the hippocampus and the thalamus, plays a central role in the pathophysiology of PTSD (Liberzon and Abelson, 2016). Furthermore, the thalamus helps in driving hippocampal functions (Cassel and Pereira de Vasconcelos, 2015). However, despite evidence of functional connectivity (FC) between the hippocampus and thalamus in healthy individuals (Cunningham et al., 2017), few studies have conducted this examination in the context of PTSD, especially for the hippocampal subfields, which play different roles in context processing (Duvernoy, 2005; Carr et al., 2010). Evaluation of the thalamus and hippocampal subfields might further illuminate the hippocampal function in context processing in people with PTSD.

Neuroimaging studies of patients with PTSD have reported sex differences (Helpman et al., 2017), mainly through comparisons between males and females who had experienced different types of trauma or who lived in different environments. However, there are still two issues that need to be addressed. First, existing studies have not adequately clarified sex differences post-trauma but before PTSD onset. Second, it remains unclear whether the reported differences between male and female patients were induced by variations in trauma types or their post-trauma living environments. Thus, a neuroimaging study on couples without PTSD who live together and have experienced the same trauma—losing their only child—could provide valuable insight.

Previous studies have shown that female patients experienced more re-experiencing symptoms and were more likely to meet the criteria for PTSD than male patients (Zlotnick et al., 2001), and parents who lost their only child also showed significant sex differences in the incidence of PTSD and brain topological properties (Yin et al., 2018; Luo et al., 2019). However, sex differences in re-experiencing symptoms and context processing with regard to the trauma of losing one’s only child and PTSD have not been investigated, and the neural basis remains ambiguous.

Therefore, in this study, conducted to explore the neural mechanism of sex differences in re-experiencing symptoms, we recruited relatively homogeneous couples without any psychiatric disorder who experienced the same trauma—the death of their only child—and analyzed FC between the hippocampal subregions and thalamus and the correlations of FC with re-experiencing symptoms. Moreover, patients with PTSD and normal controls (NCs) were recruited to explore the changing trend in FC between the sexes, to further elaborate its role between the hippocampal subregions and thalamus in couples without PTSD.

## Materials and Methods

### Participants

This study was approved by the Ethics Committee of Jiangsu University, and written informed consent was obtained from all participants, in keeping with the Declaration of Helsinki. In 2017, a small PTSD survey of Han Chinese adults who lost their only child was performed in China. The survey included 237 such parents among whom 30 couples without any psychiatric disorder were selected (non-PTSD group). The comparison between husbands and wives was conducted using the paired *t*-test, which is considered ideal for analyzing sex differences. The Structured Clinical Interview for the Diagnostic and Statistical Manual of Mental Disorders, Fourth Edition was used to ensure that these couples did not have any psychiatric disorder (First et al., 1998). The severity of symptoms, especially re-experiencing symptoms (Criterion B and items B1-5), was evaluated by the Clinician-Administered PTSD Scale (CAPS) (Blake et al., 1995).

To further illuminate sex differences, we selected 55 patients with PTSD who had also lost their only child (39 females and 16 males; PTSD group) from the total sample of 237 parents. Owing to the difficulty in recruiting enough couples where both partners had PTSD (there were only eight such couples in our database), we could only individually analyze female and male patients with PTSD to further explore the sex effect in comparison with couples without PTSD. Moreover, 50 local healthy adults (30 women and 20 men) without any trauma were chosen as the NC group.

The exclusion criteria were as follows: (1) any history of or current brain injury or other major medical or neurological conditions, (2) contraindications to magnetic resonance imaging (MRI), (3) left-handedness, (4) unavailable data, and (5) head translation of more than 1.5 mm or rotation of more than 1.5^°^ during the MRI.

### Questionnaires

Besides the CAPS, the Hamilton Depression Rating Scale (HAM-D) (Hamilton, 1960), the Hamilton Anxiety Rating Scale (HAM-A) (Hamilton, 1959), and the Mini-Mental State Examination (MMSE) (Folstein et al., 1983) were used to assess the neuropsychological situation of each participant. In addition, the Chinese Social Support Rating Scale (SSRS) (Cheng et al., 2008) and the Simple Coping Style Questionnaire (SCSQ) (Jiang et al., 2017) were used to evaluate the social support level and individual coping ability, respectively. Detailed descriptions are available in Note 1 of the Supplementary material (Supplementary Material Note 1).

### Image Acquisition

MRI data for all participants were obtained using a Philips Achieva 3.0T MRI device (Philips, Amsterdam, Netherlands). Participants were instructed to keep their eyes closed and refrain from thinking about anything during the procedure. Additionally, foam padding was used to minimize head motion. The parameters of the MRI sequences were set as follows: (1) sagittal high-resolution three-dimensional T1-weighted turbo fast echo sequence for structural data: repetition time (TR) = 9.7 ms, echo time (TE) = 4.6 ms, flip angle 9^°^, field of view (FOV) = 256 × 256 mm^2^, matrix size = 256 × 256, slice thickness = 1 mm, voxel size = 1 × 1 × 1 mm^3^, 160 slices and (2) echo planar imaging sequence for resting-state functional MRI data: TR = 2000 ms, TE = 30 ms, flip angle 90^°^, FOV = 192 × 192, voxel size = 3 × 3 × 4 mm^3^, image volumes = 230, number of axial sections per volume = 35.

### Image Processing

The image preprocessing was performed using a toolbox for Data Processing and Analysis for Brain Imaging (DPABI) (Yan et al., 2016) based on the MATLAB platform (MathWorks, Natick, MA, United States). The initial ten volumes were excluded for steady state longitudinal magnetization; the slice timing and head motion correction were conducted on all the remaining volumes. Individual T1-weighted images were co-registered to the functional images and then segmented into gray matter, white matter, and cerebrospinal fluid, and transformed into the standard Montreal Neurological Institute (MNI) space. The functional images were transformed into the MNI stereotaxic space (3 × 3 × 3 mm^3^), using the parameters of the T1-weighted image normalization, and then smoothed with an 8mm full width at half maximum (FWHM) isotropic Gaussian kernel. The mean signals from cerebrospinal fluid and white matter were regressed out. The imaging data were also temporally filtered (bandpass: 0.01–0.1 Hz) (Qi et al., 2020).

After preprocessing, the FC between the hippocampal subfields (four regions in each hemisphere: the cornu ammonis (CA1, CA2, and CA3) and dentate gyrus) and the thalamus was analyzed within each group (female vs. male) and between the three groups (non-PTSD, PTSD, and NC). The cytoarchitectonically probabilistic maps from the JuBrain Cytoarchitectonic Atlas in the SPM Anatomy Toolbox (Eickhoff et al., 2005) were used to define the hippocampal subregions in each hemisphere (Supplementary Figure 1). The process of analyzing the FC between the hippocampal subfields and thalamus included the computation of the average time series, the correlation coefficients, and the resting-state fc map (Qi et al., 2020) (Supplementary Material Note 2 in detail).

### Statistical Analyses

The data were analyzed using SPSS version 25 (IBM Corp., Armonk, NY, United States). Between-group comparisons of the demographic characteristics and neuropsychological scores were performed using analysis of variance (ANOVA) statistics for the continuous variables.

With regards to the wives and husbands in the non-PTSD group, the paired *t*-test was used to compare differences in FC between hippocampal subfields and the thalamus using DPABI. Significant clusters were identified using the Gaussian Random Field at corrected *p* < 0.05, which corresponded to a voxel *p* < 0.01 and a cluster level with *p* < 0.05. Then, the correlations of the FC with the CAPS scores in the non-PTSD group were assessed using partial correlations, accounting for age and educational level.

For the NC and PTSD groups, based on the significant results in the paired *t*-test and correlation conducted with the non-PTSD group, FC was reprocessed in the DPABI using the mask, after which the values were extracted from single-subject FC maps in all participants. Then the 2 (male and female) × 3 (HC, non-PTSD, and PTSD) ANOVA investigating the interaction between sex and diagnosis was quoted. Additionally, the *post hoc* analysis (sex differences in FC within the NC and PTSD groups, as well as between the three groups) was performed. Results were considered significant at corrected *p* < 0.05 (using the false discovery rate correction for multiple comparisons).

## Results

### Demographic Data and Clinical Comparisons

Between the wives/women and husbands/men in every group, there were no significant differences in age, educational level, duration of trauma, and scores on the SSRS, SCSQ, MMSE, and total CAPS (*p* > 0.05). However, compared with husbands without PTSD, wives without PTSD had higher scores on the HAM-A and Criterion B and items B1 and B4 on the CAPS (*p* < 0.05). Additionally, HAM-D scores in female patients with PTSD were higher than in male patients with PTSD (Table 1). Among the NC, non-PTSD, and PTSD groups, no significant differences were found in age, educational level, and MMSE scores in male or female participants. SSRS and SCSQ scores were not significantly different between the PTSD and non-PTSD groups for both female and male participants (Supplementary Table 1).

**TABLE 1:** Demographic data and clinical comparisons between male and female within groups.

	**PTSD**				**Non-PTSD**				**NC**			
	**Male (16)**	**Female (39)**	***T***	***P***	**Husband (30)**	**Wife (30)**	***T***	***P***	**Male (20)**	**Female (30)**	***T***	***P***
Age (years)	59.06 + 6.51	56.95 + 5.04	1.30	0.20	59.33 + 5.86	56.63 + 5.71	1.81	0.076	55.00 + 6.92	56.17 + 5.31	0.674	0.50
Years of education	6.38 + 3.61	6.54 + 4.40	0.13	0.90	7.80 + 2.44	6.40 + 3.67	1.74	0.087	7.35 + 4.51	7.33 + 3.60	0.01	0.99
MMSE	26.00 + 2.94	25.77 + 3.28	0.24	0.81	26.97 + 2.16	25.83 + 2.63	1.83	0.073	27.95 + 2.26	27.03 + 2.46	1.33	0.19
HAMD	12.88 + 5.05	17.18 + 6.96	2.24	***0.029***	5.43 + 4.42	7.31 + 4.54	1.61	0.11				
HAMA	10.19 + 5.85	13.51 + 6.74	1.72	0.091	4.17 + 3.51	6.48 + 3.80	2.43	***0.018***				
Time duration (mo)	54.06 + 44.93	61.33 + 50.31	0.50	0.62	115.87 + 63.20	115.83 + 63.22	0.002	0.998				
CAPS Score	45.94 + 12.40	47.38 + 12.55	0.39	0.70	13.87 + 9.50	19.13 + 12.01	1.88	0.065				
B	15.81 + 6.00	16.51 + 6.13	0.39	0.70	5.57 + 4.93	10.33 + 6.89	3.08	***0.003***				
B1	5.56 + 1.86	5.85 + 0.88	0.77	0.44	2.20 + 2.04	4.20 + 2.07	3.76	***0.000***				
B2	2.38 + 2.50	2.64 + 2.98	0.31	0.76	0.63 + 1.47	1.07 + 2.08	0.93	0.36				
B3	0.81 + 1.80	0.85 + 1.74	0.065	0.95	0.20 + 1.10	0.40 + 1.25	0.66	0.51				
B4	4.69 + 1.25	4.49 + 1.70	0.43	0.67	2.00 + 1.58	3.30 + 1.88	2.90	***0.005***				
B5	2.38 + 2.68	2.69 + 2.26	0.45	0.66	0.53 + 1.14	1.37 + 2.08	1.93	0.059				
SSRS												
SSRS_total	37.88 + 6.17	40.05 + 7.35	1.04	0.30	39.27 + 6.34	41.03 + 6.89	1.03	0.31				
Objective support	11.06 + 2.32	12.45 + 2.72	1.79	0.08	12.43 + 2.27	13.60 + 2.44	1.91	0.60				
Subject support	21.50 + 4.41	21.36 + 3.70	0.12	0.90	21.80 + 3.68	21.53 + 4.07	0.27	0.79				
Utility of support	5.31 + 1.92	5.74 + 2.04	0.73	0.47	5.03 + 1.99	5.90 + 2.04	1.67	0.10				
SCSQ												
Active	19.38 + 5.49	17.85 + 6.77	0.80	0.43	20.57 + 5.59	20.07 + 6.38	0.32	0.75				
Negative	10.63 + 3.10	9.69 + 2.83	1.08	0.29	11.23 + 3.75	10.43 + 3.51	0.85	0.40				
Copying tendency	8.75 + 5.34	8.15 + 6.24	0.34	0.74	9.33 + 6.04	9.63 + 5.52	0.20	0.84				

**Values are expressed as mean ± SD. PTSD, post-traumatic stress disorder; NC, normal control; CAPS, Clinician-Administered PTSD Scale, SSRS, Social Support Rating Scale; SCSQ, Simple Coping Style Questionnaire; MMSE, Mini-Mental State Examination.* The bold values mean *P* < 0.05.*

### FC and Correlation Between Wives and Husbands in the Non-PTSD Group

**FIGURE 1:**
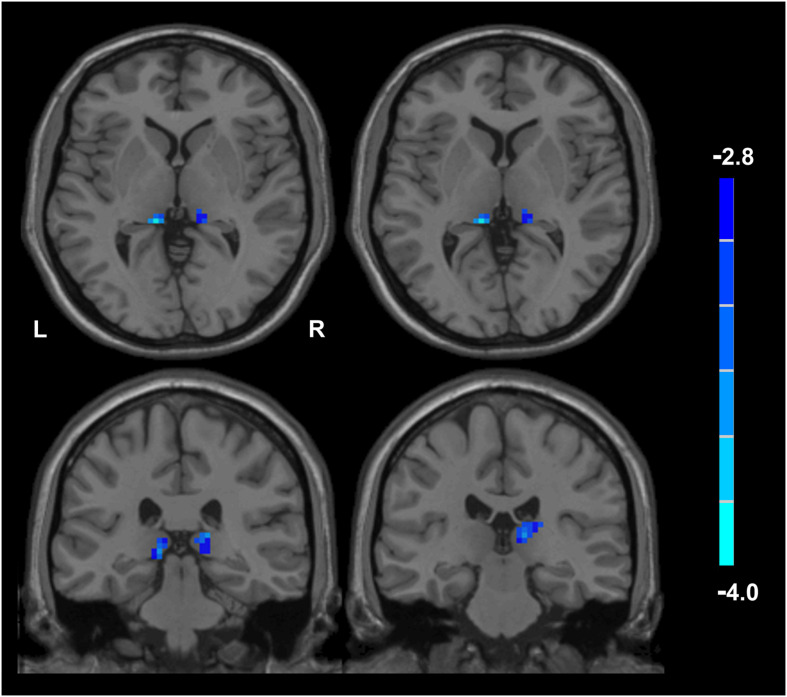
Functional connectivity (FC) between the wives and husbands in the non-PTSD group. Compared with the non-PTSD husbands, the non-PTSD wives had weaker FC between the right hippocampal CA3 and the bilateral thalamus (*p* < 0.05, corrected). PTSD, post-traumatic stress disorder; CA, Cornu Ammonis.

**FIGURE 2:**
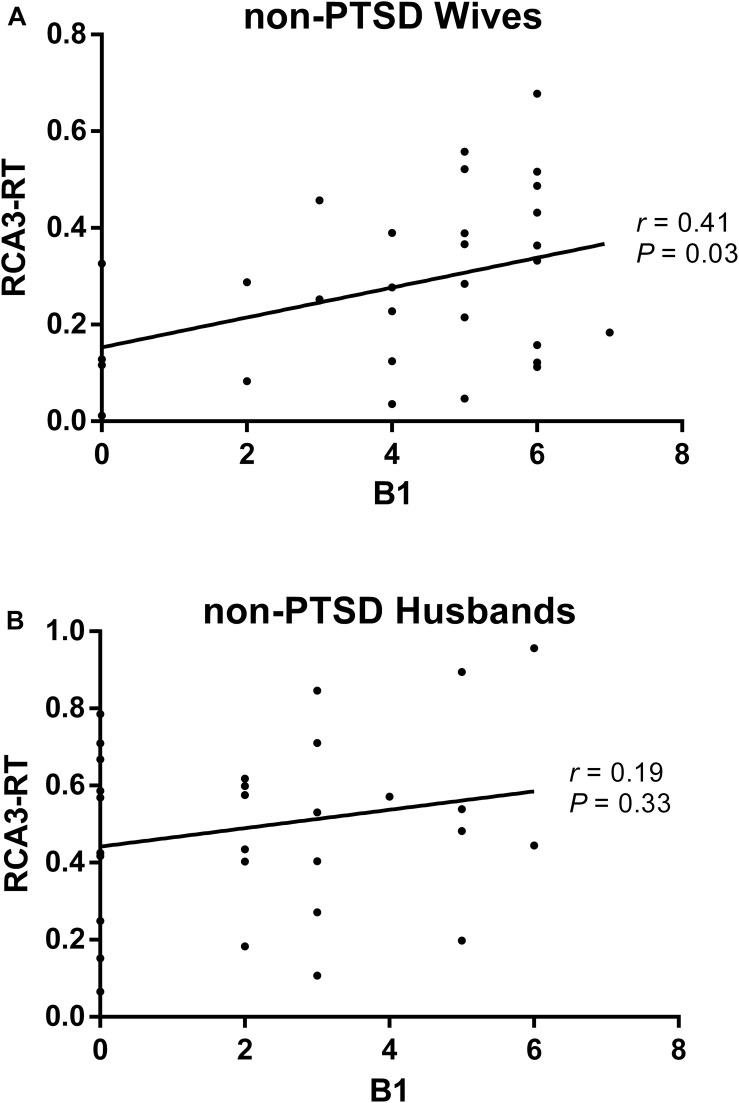
The correlation between the re-experiencing symptom and the abnormal functional connectivity (FC) in the non-PTSD wives **(A)** and husbands **(B)**. Only the correlation between the RCA3-RT FC and B1 score was significant (*r* = 0.41, *P* = 0.03) in the non-PTSD wives, but not in the non-PTSD husbands. RCA3-RT FC, the functional connectivity between right hippocampal CA3 and right thalamus; CA, Cornu Ammonis.

Compared with husbands without PTSD, wives without PTSD had weaker FC between the right hippocampal CA3 and the right thalamus (RCA3-RT), as well as FC between the right hippocampal CA3 and the left thalamus (Figure 1 and Table 2). Based on the clinical comparisons in Table 1, the correlations between the B, B1, and B4 scores and the abnormal FC were investigated in the group without PTSD. Only the correlation between the RCA3-RT FC and B1 score was significant in wives without PTSD (*r* = 0.41, *p* = 0.03), but not in husbands (Figure 2).

**TABLE 2:** FC of right hippocampal CA3 between wife and husband of non-PTSD (*p* < 0.05, corrected).

**Brain regions**	**BA**	**MNI coordinates (mm)**	**Voxel number**	**Statistic values**
		**(x, y, z)**		
Right thalamus	27	12, −27, 9	39	3.97
Left thalamus		−12, −33, 3	10	3.91

**FC, functional connectivity; CA, cornu ammonis; BA, Brodmann area, MNI, Montreal Neurologic Institute; non-PTSD, non-post-traumatic stress disorder*.*

### Comparisons of FC and Correlation in Other Groups

**FIGURE 3:**
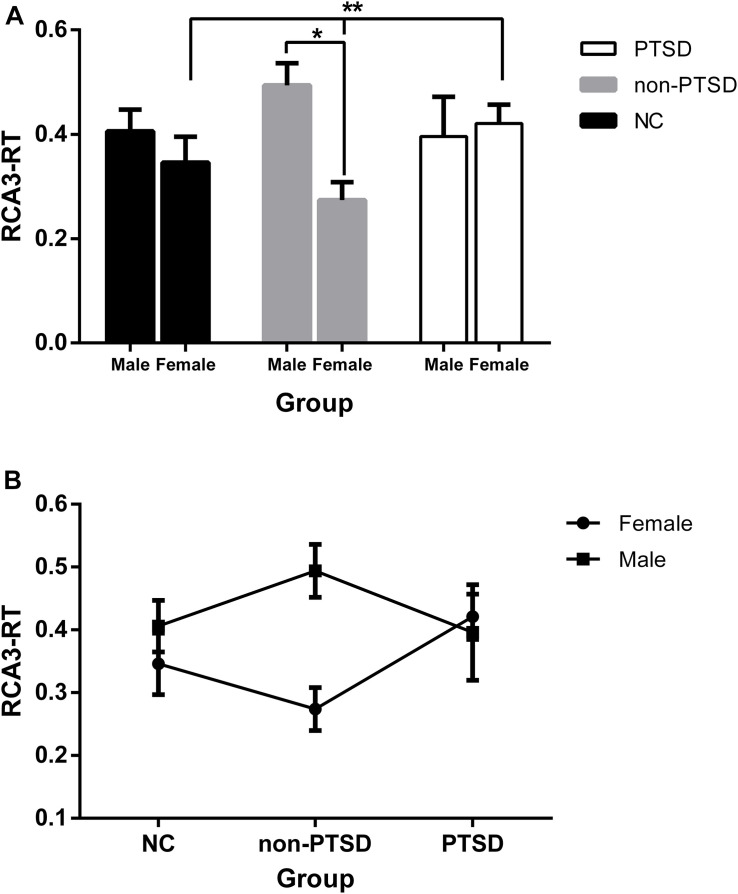
Functional connectivity (FC) between the female and male within and among the groups adjusting for the effects of age and education levels. **(A)** Except the non-PTSD group (*), neither the NC nor the PTSD group, the RCA3-RT FC was not significantly different between the male and female. Additionally, in the female participants, the RCA3-RT FC was significantly different among the NC, non-PTSD, and PTSD groups (**); **(B)** the dynamic changes in the three groups in the male or female. RCA3-RT, right hippocampal CA3 and the right thalamus; CA, Cornu Ammonis; PTSD, post-traumatic stress disorder; NC, normal control; *, **P < 0.05.

Through comparisons of the values extracted from single-subject FC maps, the main effect of sex was significant (*F* = 5.76, *p* = 0.018), as well as the diagnosis-by-sex interaction effect (*F* = 4.49, *p* = 0.013). Additionally, only the RCA3-RT FC in female participants was markedly different among the NC, non-PTSD, and PTSD groups (*F* = 3.61, *p* = 0.031), and the RCA3-RT FC between wives without PTSD and female patients with PTSD was remarkably different (*p* = 0.009). However, the RCA3-RT FC was not significantly different between men and women in the NC or PTSD groups (Figure 3A; NC: *F* = 0.44, *p* = 0.51; PTSD: *F* = 0.27, *p* = 0.61). Moreover, the correlation between the RCA3-RT FC and B1 score was not significant in male and female participants with PTSD (*p* > 0.05).

As depicted in Figure 3B, the change tendency of the RCA3-RT FC in female participants showed that compared with their NC counterparts, FC in wives without PTSD declined, and significantly rose in female patients with PTSD. Among male participants, compared with their NC counterparts, FC in husbands without PTSD increased, and declined in male patients with PTSD.

### Supplementary Analyses

The HAM-D and HAM-A scores were significantly different between some groups (Table 1), along with age and educational level. Thus, they were added as covariates in the correlation analysis of the non-PTSD group. Like the above results, the correlation between the RCA3-RT FC and B1 score was significant (*r* = 0.43, *p* = 0.027) only in wives without PTSD (Supplementary Figure 2).

Additionally, based on the FC result, to eliminate the possible confounding effect of hippocampal structural alteration on functional abnormality, voxel-based morphometry analysis was used to analyze the CA3 volume in a manner similar to prior PTSD studies (Chen and Etkin, 2013; Qi et al., 2020). We found that CA3 volume was not significantly different between the male and female participants within and across the three groups (Supplementary Tables 2, 3).

To minimize the confounding effect of head motion (Yan C. et al., 2013; Yan C. G. et al., 2013), the Friston 24-parameter model was used to regress out head motion effects (Friston et al., 1996). The mean framewise displacement was also added as a covariate. The RCA3-RT FC was also significantly different among the NC, non-PTSD, and PTSD groups only in female participants (*F* = 3.73, *p* = 0.028). The correlation between the RCA3-RT FC and B1 score was also significant only in wives without PTSD (*p* = 0.04).

## Discussion

This is the first neuroimaging study to explore the sex effect of the trauma by using the couples who live together and have experience of the same trauma, which was the important advantage of this study. In the present study, we investigated the FC between the hippocampal subfields and thalamus and the association between FC and re-experiencing symptoms in wives and husbands without PTSD who lost their only child, as well as in parents with PTSD. We found that wives who had lost their only child but did not have PTSD showed worse re-experiencing symptoms (B1), which was associated with lower FC between the right CA3 and the thalamus, than their husbands. Importantly, lower FC between CA3 and the thalamus in wives may reflect a protective factor against the development of PTSD.

Re-experiencing symptoms include sudden, unwanted memories of the trauma, nightmares, and even distortions of experience that can make an individual feel as if the event is recurring (Schnurr et al., 2009). Thus, long-term and higher re-experiencing symptoms may result in a higher likelihood of trauma-exposed individuals developing PTSD. In the present study, despite similar social support levels and individual coping ability, wives who had lost their only child but did not have PTSD showed more serious re-experiencing symptoms than their husbands did. Thus, these wives might be at greater risk for the development of PTSD. However, many wives had not developed PTSD despite the presence of re-experiencing symptoms for several years (the average duration since the loss of the only child was more than 10 years).

The hippocampus plays a critical role in contextual learning and memory (Holland and Bouton, 1999). It establishes new memory representations, minimizes overlapping of previous memories, and also helps retrieve old memories based on partial information (Carr et al., 2010; Liberzon and Abelson, 2016). These processes include two abilities: (1) pattern separation: diminishing the similarity between two similar memories and (2) pattern completion: recalling a memory based on a partial cue (Yassa and Stark, 2011). The CA3 is thought to be involved in both processes. Compared with their husbands, wives without PTSD showed lower FC between the right CA3 and the thalamus. Moreover, the correlational analysis revealed that the RCA3-RT FC was positively associated with item B1 (recurrent and intrusive distressing recollections of the event) only in wives without PTSD. This suggested that the alteration of the hippocampal subfield FC is related to worse re-experiencing symptoms in wives who have lost their only child.

To further explore the role of the abnormal RCA3-RT FC in wives who had lost their only child but did not have PTSD, both male and female NC, non-PTSD, and PTSD groups were investigated. The differences in RCA3-RT FC between healthy female and male participants were not significant. This implied that the differences in RCA3-RT FC between wives and husbands without PTSD resulted from re-experiencing symptoms and not sex. Additionally, from the analysis of the female participants in the three groups, we found that RCA3-RT FC in wives without PTSD was lower than in healthy individuals and female patients with PTSD. This suggested that the decrease in the RCA3-RT FC may be a protective factor against the development of PTSD in wives who have lost their only child. Furthermore, the positive association between re-experiencing symptoms (B1) and RCA3-RT FC implied that lower RCA3-RT FC might be an indication of more robust protective ability in wives without PTSD. The neurocircuitry models of PTSD showed that the hippocampus and prefrontal cortex could inhibit hyperactivity in the amygdala to prevent the development of PTSD (Pitman et al., 2012), and the hippocampus and prefrontal cortex are also included in the context processing circuitry (Liberzon and Abelson, 2016). Furthermore, the hippocampal-prefrontal communication involves the thalamus, which participates in the consolidation of enduring memories at the system level (Cassel and Pereira de Vasconcelos, 2015). Thus, a lower RCA3-RT FC, which implied a reduction in the communication between the hippocampal subfield-CA3 and thalamus, suggested that after the severe trauma of losing their only child, the consolidation of traumatic enduring memories was weakened in wives without PTSD; this, in turn, reduced re-experiencing symptoms and protected them from PTSD.

Our study had several limitations. First, based on the key role and the interrelation of the hippocampal subfields and the thalamus in the context processing, we focused only on the FC between the hippocampal subfields and the thalamus in the present study. However, a deeper analysis of brain network alteration during the development of PTSD may be beyond the scope of the current study. Other aspects of FC, such as the default mode and executive control networks, would be examined in future studies. Second, the cross-sectional design of the study prevented us from ascertaining how the RCA3-RT FC changed over time in couples without PTSD. Third, although it is beneficial to control other variances, the trauma examined in this study was very specific. Hence, any attempt to generalize the results to other traumatic events must be undertaken with caution. Fourth, the sample size of the non-PTSD couples was small, and the samples of the PTSD and NC groups were not couples. Thus, future studies must recruit more couples to enhance the reliability of our study.

In conclusion, our study revealed that wives who lost their only child but did not develop PTSD had more serious re-experiencing symptoms than their husbands. The severity of the re-experiencing symptoms was associated with the FC changes between the hippocampal subregions and the thalamus only in these wives. Importantly, our results suggest a potential protective role of the low level of the RCA3-RT FC in wives without PTSD.

## Data Availability Statement

The datasets generated for this study are available on request to the corresponding authors.

## Ethics Statement

The studies involving human participants were reviewed and approved by the Medical Research Ethics Committee of Jiangsu University. The patients/participants provided their written informed consent to participate in this study.

## Author Contributions

ZC, GL, and RQ: experiment design. YFL and ZQ: manuscript writing. YL: image processing and statistical analyses. YW and HS: image acquisition. LZ, LL, and XZ: participant recruitment. All authors read and approved the final manuscript.

## Conflict of Interest

The authors declare that the research was conducted in the absence of any commercial or financial relationships that could be construed as a potential conflict of interest.
