# Supplementary material for: Sex Differences in Re-experiencing Symptoms Between Husbands and Wives Who Lost Their Only Child in China: A Resting-State Functional Connectivity Study of Hippocampal Subfields
Source: Front Hum Neurosci. 2021 Apr 28;15:655044. doi: 10.3389/fnhum.2021.655044 (PMC8113639; doi:10.3389/fnhum.2021.655044)
Supplement: Supplementary file 1 [file Data_Sheet_1.DOCX]

**Supplementary Material**

1. **Supplementary Note 1: Measures of Neuropsychological Tests**
2. **Supplementary Note 2: The detailed process of the hippocampal subfields functional connectivity**
3. **Supplementary Tables**
4. **Supplementary Figures**
5. **Supplementary Note 1: Measures of Neuropsychological Tests**

All participants were assessed with neuropsychological tests included: Hamilton Depression (HAMD) ([1](#_ENREF_1)) and Hamilton Anxiety (HAMA) ([2](#_ENREF_2)) rating scales, Mini-Mental State Examination (MMSE) ([3](#_ENREF_3)), Chinese Social Support Rating Scale (SSRS), containing subjective and objective support, and the utility of support ([4](#_ENREF_4)), and individual Simple Coping Style Questionnaire (SCSQ), including active and negative coping score, and the coping tendency score ([5](#_ENREF_5)). For detail, the SSRS contains three subscales of social support: subjective support, objective support and the utility of support. Subjective support reflects the perceived interpersonal network that a person could bank on (4 items with scores ranging from 8-32). Objective support refers to the actual support a person gained in the past (3 items with scores ranging from 1-22). The utility of support reflects personal active seeking of various social supports (3 items with scores ranging from 3-12). The total SSRS scores are the sum of all three subscales, and higher scores (ranging from 12-66) imply stronger social support. The SCSQ contains assessments of active (12 items) and negative coping (8 items), respectively. The scale of each item uses 4-level Likert score standards, in which ‘3’ stands for regular use, while ‘0’ stands for no use. Then the scores for active and negative coping are measured independently, and a higher score indicates the inclination to adopt the corresponding coping style, while the coping tendency scores are defined as the active coping scores minus the negative coping scores.

1. **Supplementary Note 2: The detailed process of the hippocampal subfields functional connectivity**

For each participant, the average time series across all voxels of each hippocampal subregion was computed as a reference time course separately, and then correlated with the time series of the rest of the brain. The correlation coefficients were converted to z values using Fisher’s r-to-z transformation to standardize the statistical analysis. Thus, the whole brain resting-state functional connectivity map of each hippocampal subregion was generated for each participant, and was then used to identify regions showing functional connectivity differences.

1. **Supplementary Tables**

**Table 1 Demographic Data and Clinical Comparisons in male or female subjects**

|  |  | Male | | | |  |  | Female | | | |
| --- | --- | --- | --- | --- | --- | --- | --- | --- | --- | --- | --- |
|  | NC(20) | non-PTSD (30) | PTSD(16) | *F* | *P* |  | NC(30) | non-PTSD (30) | PTSD(39) | *F* | *P* |
| **Age(y)** | 55.00+6.92 | 59.33+5.86 | 59.06+6.51 | 3.11 | 0.051 |  | 56.17+5.31 | 56.63+5.71 | 56.95+5.04 | 0.18 | 0.83 |
| **Years of education** | 7.35+4.51 | 7.80+2.44 | 6.38+3.61 | 0.89 | 0.42 |  | 7.33+3.60 | 6.40+3.67 | 6.54+4.40 | 0.50 | 0.61 |
| **MMSE** | 27.95+2.26 | 26.97+2.16 | 26.00+2.94 | 2.96 | 0.59 |  | 27.03+2.46 | 25.83+2.63 | 25.77+3.28 | 1.96 | 0.15 |
| **Time Duration(mo)** |  | 115.87+63.20 | 54.06+44.93 | 3.47 | 0.001 |  |  | 115.83+63.22 | 61.33+50.31 | 3.99 | 0.00 |
| **SSRS** |  |  |  |  |  |  |  |  |  |  |  |
| **SSRS_total** |  | 39.27+6.34 | 37.88+6.17 | 0.72 | 0.48 |  |  | 41.03+6.89 | 40.05+7.35 | 0.56 | 0.57 |
| **Objective support** |  | 12.43+2.27 | 11.06+2.32 | 1.93 | 0.06 |  |  | 13.60+2.44 | 12.45+2.72 | 1.82 | 0.07 |
| **Subject support** |  | 21.80+3.68 | 21.50+4.41 | 0.25 | 0.81 |  |  | 21.53+4.07 | 21.36+3.70 | 0.18 | 0.86 |
| **Utility of support** |  | 5.03+1.99 | 5.31+1.92 | 0.46 | 0.65 |  |  | 5.90+2.04 | 5.74 +2.04 | 0.32 | 0.75 |
| **SCSQ** |  |  |  |  |  |  |  |  |  |  |  |
| **Active** |  | 20.57+5.59 | 19.38+5.49 | 0.69 | 0.49 |  |  | 20.07+6.38 | 17.85+6.77 | 1.38 | 0.17 |
| **negative** |  | 11.23+3.75 | 10.63+3.10 | 0.56 | 0.58 |  |  | 10.43+3.51 | 9.69+2.83 | 0.97 | 0.33 |
| **Copying tendency** |  | 9.33+6.04 | 8.75+5.34 | 0.32 | 0.75 |  |  | 9.63+5.52 | 8.15+6.24 | 1.03 | 0.31 |

Values are expressed as mean ± SD. PTSD: post-traumatic stress disorder; NC: normal control; CAPS: Clinician-Administered PTSD Scale; SSRS: Social Support Rating Scale; SCSQ: Simple Coping Style Questionnaire; MMSE: Mini-Mental State Examination;

**Table 2 Comparisons of the Right CA3 volume between male and female within groups**

|  | PTSD | | | |  | non-PTSD | | | |  | NC | | | |
| --- | --- | --- | --- | --- | --- | --- | --- | --- | --- | --- | --- | --- | --- | --- |
|  | Male | Female | *F* | *P* |  | Husbands | Wives | *F* | *P* |  | Male | Female | *F* | *P* |
| Right CA3 | 0.37+0.043 | 0.35+0.042 | 0.98 | 0.33 |  | 0.37+0.044 | 0.36+0.036 | 0.57 | 0.46 |  | 0.36+0.036 | 0.35+0.036 | 0.48 | 0.49 |

Values are expressed as mean ± SD. PTSD: post-traumatic stress disorder; NC: normal control;

**Table 3 Comparisons of the Right CA3 volume of male and female among groups**

|  | Female | | | | |  | Male | | | | |
| --- | --- | --- | --- | --- | --- | --- | --- | --- | --- | --- | --- |
|  | NC | non-PTSD | PTSD | *F* | *P* |  | NC | non-PTSD | PTSD | *F* | *P* |
| Right CA3 | 0.35+0.036 | 0.36+0.036 | 0.35+0.042 | 1.46 | 0.24 |  | 0.36+0.036 | 0.37+0.044 | 0.37+0.043 | 0.69 | 0.51 |

Values are expressed as mean ± SD. PTSD: post-traumatic stress disorder; NC: normal control;

1. **Supplementary Figures**


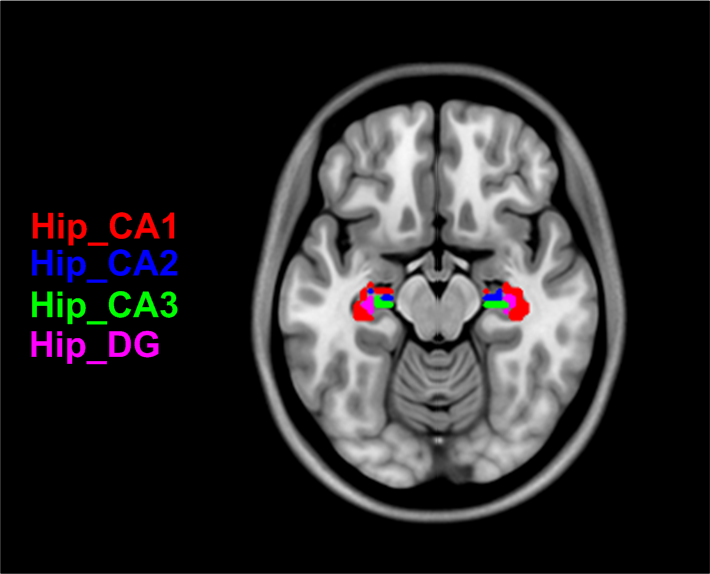


**Figure 1**. Illustration of the seed regions of interest (ROI) of the hippocampal subfields

Bilateral CA1, CA2, CA3, and DG are used in the present functional connectivity analyses. CA = cornu ammonis; DG = dentate gyrus.





**Figure2:** The correlation between the reexperiencing symptom (B1) and the abnormal functional connectivity (FC) in the non-PTSD wives and husbands adjusting for the effects of age, education levels, HAMD, and HAMA scores, and the differences were not significantly changed compared with those adjusting for the effects of age and education levels. Only the correlation between the RCA3-RT FC and B1 score was significant (*P*=0.027) in the non-PTSD wives, but not in the non-PTSD husbands. RCA3-RT FC: the functional connectivity between right hippocampal CA3 and right thalamus; CA: Cornu Ammonis.

**REFERENCES**

1. Hamilton M. A rating scale for depression. *J Neurol Neurosurg Psychiatry* 1960; **23**(1)**:** 56.

2. Hamilton M. The assessment of anxiety states by rating. *Br J Med Psychol* 1959; **32**(1)**:** 50-55.

3. Folstein MF, Robins LN, Helzer JE. The Mini-Mental State Examination. *Arch Gen Psychiatry* 1983; **40**(7)**:** 812.

4. Cheng Y*, et al*. Social support plays a role in depression in Parkinson's disease: a cross-section study in a Chinese cohort. *Parkinsonism Relat Disord* 2008; **14**(1)**:** 43-45.

5. Jiang X-R, Du J-J, Dong R-Y. Coping style, job burnout and mental health of university teachers of the millennial generation. *EURASIA J Math, Sci Tech Ed* 2017; **13**(7)**:** 3379-3392.
